# Concentrations of Circulating Irisin and Myostatin in Race and Endurace Purebred Arabian Horses—Preliminary Study

**DOI:** 10.3390/ani10122268

**Published:** 2020-12-01

**Authors:** Sylwester Kowalik, Anna Wiśniewska, Witold Kędzierski, Iwona Janczarek

**Affiliations:** 1 Department of Animal Physiology, Faculty of Veterinary Medicine, University of Life Sciences in Lublin, ul. Akademicka 12, 20-033 Lublin, Poland; sylwester.kowalik@up.lublin.pl; 2 Department of Horse Breeding and Use, Faculty of Animal Sciences and Bioeconomy, University of Life Sciences in Lublin, ul. Akademicka 13, 20-950 Lublin, Poland; iwona.janczarek@up.lublin.pl; 3 Department of Biochemistry, Faculty of Veterinary Medicine, University of Life Sciences in Lublin, ul. Akademicka 12, 20-033 Lublin, Poland; witold.kedzierski@up.lublin.pl

**Keywords:** hormones, irisin, myostatin, exercise, endurance, racehorses

## Abstract

**Simple Summary:**

Irisin and myostatin are regulatory proteins produced by muscle cells. The aim of the study was to evaluate the effect of exercise on plasma irisin and myostatin concentrations in horses in different types of training (speed versus endurance). To find out, we tested 20 Arabian horses, submitted to the two different equestrian disciplines, and consequently different training regimes. The first group of horses realized a short-term, high-speed bout of exercise whereas the second group of horses were submitted to long-lasting, endurance effort. The obtained results showed that the single bout of exercise induced an increase in plasma myostatin concentration. Plasma irisin level decreased during the race season in racehorses. This means that irisin and myostatin may play a regulatory role in the maintenance of the energy balance processes.

**Abstract:**

Skeletal muscle is considered to be the largest endocrine organ determining the maintenance of energy homeostasis. Adaptive changes in skeletal muscles in response to physical exercise influence the production as well as secretion of myokines, which are bioactive factors that play a crucial role in energy expenditure processes. The aim of the study was to investigate the impact of two different types of exercise on the circulating level of two of these, myostatin and irisin, in trained horses. Twenty purebred Arabian horses were involved in the study: 10 three-year-old horses trained on the racetrack and 10 endurance horses aged 7.4 ± 1.9 years. The horses from both groups were regularly trained throughout the entire season, during which they also participated in Polish National competitions. To assess the influence of the training sessions on plasma myostatin and irisin concentrations, blood samples taken at rest and 30 min after the end of exercise were analyzed. In the studied horses, the single bout of exercise did not influence plasma irisin but induced an increase in plasma myostatin concentration. In racehorses, plasma irisin concentration decreased with the length of the training season. Plasma myostatin was higher in endurance horses than in three-year-old racehorses. Lack of exercise-induced fluctuation in circulating irisin in studied horses suggests that myostatin released in response to exercise provides a negative feedback signal to irisin release.

## 1. Introduction

Exercise is essential for improving both the general musculoskeletal fitness and athletic performance of sport horses [1,2,3]. The high exercise capacity required during exercise tests is achieved largely via long and arduous training processes [4,5,6]. A properly maintained training regime improves the efficiency of the musculoskeletal system and ensures energy homeostasis during intensive muscular work [7]. This is particularly important as skeletal muscle is responsible for about 90% of energy expenditure during exercise [8]. In such conditions, the maintenance of a functional balance between energy production and expenditure demands the engagement of local and/or systemic endocrine mechanisms. Muscle tissue is considered to be the largest endocrine organ determining the maintenance of energy homeostasis [9,10,11]. This is based on detailed analyses of the secretory function of muscle tissue, which revealed that a complex of over 300 protein agents are actively secreted by skeletal myocytes [12,13]. These muscle-derived biologically active proteins are collectively referred to as myokines, tissue hormones that interact with numerous target sites [12]. Adaptive changes in skeletal muscles to physical activity result in adjustments of the production and secretion of these bioactive factors into the bloodstream. Hence, they should be considered as specific messengers that allow communication between muscles and other organs such as adipose tissue, liver, bone, and the nervous system in order to produce the beneficial effects of exercise at the whole-body level [14,15,16,17]. Recent advances have demonstrated that myokines are able to regulate lipid mobilization from adipose tissue, liver endogenous glucose production, and insulin secretion by beta pancreatic cells [18,19]. Accordingly, intense physical activity might influence the rate of both lipid and glucose metabolism, both of which are highly important in the maintenance of the energy balance [20]. In humans, studies on this topic have been conducted for years but in equine exercise physiology the precise actions of myokines are still not fully understood [21]. The latest human research has focused on myostatin and irisin as potentially important new players in energy homeostasis [15,22,23,24,25]. 

Myostatin, also known as growth differentiation factor 8 (GDF8), a member of the transforming growth factor-β superfamily, is a powerful regulator of skeletal muscle mass in mammals [26,27]. The main function of myostatin is the negative regulation of muscle growth by inhibiting myoblast proliferation and differentiation [28,29]. In experimental animals, deletion of the myostatin gene induces a dramatic and widespread increase in skeletal muscle mass due to both muscle hypertrophy and hyperplasia [30]. Although its main function is related to controlling the quantity of skeletal muscle, myostatin plays an important role in the modification of energy expenditure [22,24,30]. It is known that myostatin deficiency promotes browning of white adipose tissue (WAT), and as a consequence, increases total energy expenditure. Furthermore, elevated energy expenditure inhibits fat gain while simultaneously increasing lean body mass [31]. Finally, as a consequence of these changes, myostatin can improve glucose uptake in skeletal muscle due to a reduction in insulin resistance [32]. 

Although the origin and metabolism of myostatin are more or less defined, its function appears to be dependent on a network of interactions with other endocrine proteins. Among these, irisin has recently been indicated as a factor that may play an essential role in this network [33]. This muscle-derived peptide is recognized as a product of the FNDC5 muscle gene, which is stimulated by the transcriptional co-activator PPAR-γ co-activator-1α (PGC1-α) [34]. Currently, it is widely believed that the role of FNDC5 and irisin is to increase energy expenditure by stimulating the browning of WAT. As one of the main sites of energy storage, WAT releases a variety of active peptides that modulate whole-body metabolism, whereas brown adipose tissue (BAT) increases energy expenditure, thus creating a negative energy balance [35]. The irisin-dependent effect of browning WAT is mediated by the expression of uncoupling protein 1 (UCP1), and the changes that accompany this process include stimulation of fatty acid oxidation, improvement of glucose intolerance and finally increased energy expenditure [34,36,37]. Therefore, irisin appears to play the role of a powerful messenger in crosstalk between muscle and adipose tissue [20,37,38]. It has also been reported that in humans and mice, FNDC5 expression is induced by physical exercise. A study by Brenmoehl et al. [39] and Kraemer et al. [40] revealed a transient increase in circulating irisin in response to moderate and acute physical exercise. However, the conclusions of other studies called this statement into question [41,42,43]. Taken together, it appears that myostatin and irisin are capable of modulating and controlling whole-body energy expenditure, but via different regulatory pathways [11,32,44]. The results of a study by Dong et al. [35] revealed that inhibition of myostatin caused the conversion of WAT to BAT while stimulating energy expenditure. In turn, irisin was recognized as one of the main mediators in this muscle-to-fat cross talk mechanism, which stimulates the browning process of WAT by enhancing the expression of UCP1 coupled with an increase in PGC1α. All these potential effects of both irisin and myostatin prompted us to undertake a study on the influence of exercise on plasma levels of these myokines in trained horses.

The exercise intensity and the level of work-load of horses can be estimated using chosen biochemical indicators. The exercise-induced changes in these indicators reflect a relative workload in horses. Generally, for this purpose, lactic acid (LA) is used in racehorse studies because LA increases during short-time intense exercise whereas long-lasting effort induces an increase in plasma cortisol concentration [45]. In endurance horses, an increase in plasma proteins concentration reflects a dehydration state, and an increase in indicator enzymes, like aspartate aminotransferase (AST) and lactate dehydrogenase (LDH), reflect muscle damage that can occur during endurance effort [2,45,46]. 

The aim of the study was to evaluate the effect of exercise on changes in plasma myostatin and irisin concentrations in horses in different types of training (speed versus endurance).

## 2. Materials and Methods

### 2.1. Horses

The experiment was carried out with the approval of the Local Ethics Review Committee for Animal Experiments in Lublin (reference number: 45/2017, approval date: 22 May 2017) and was conducted according to European Community regulations.

The study was conducted using trained purebred Arabian horses. Two groups of horses were studied: Racehorses and endurance horses that were supposed to be on the standard performance level for middle racing or endurance distances. For both groups, the study was carried out during one training season, lasting in the country, where the study was performed, from March to November (and from December to February lasts a winter rest). 

The first group comprised 10 racehorses aged 3 years old, with an equal number of stallions and mares. Throughout the racing season, the horses were housed and fed in the manner recommended for racehorses. The horses were kept individually in spacious boxes with free access to water and mineral salt blocks. In accordance with their needs (i.e., depending on workload and body condition), each horse received an individually calculated ration of hay, oats, and commercial concentrate formulated for equine athletes. For example, in July, the diet included an average of 0.27 MJ/kg body weight (BW) per day of digestible energy and 2.5 g/kg BW/day of digestible protein, distributed three times per day (06.00 am, 12.00 am, and 06.00 pm). The racehorses were trained and competed in official races on the Służewiec Horse Race Track (Warsaw, Poland). They started training in the middle of March, and from the middle of May they were entered in official races at least once a month. All training procedures for studied horses were performed under the care of one professional trainer. The typical daily training routine consisted of three phases: 10 min of walking as a warm-up, 10 min of trot or canter, and galloping over the distance of 1200 m at a speed between 6 and 12 m/s, according to horse performance and the trainer’s instructions (detailed data are presented in Table 1). Every training session was completed by putting the horses on an automatic horse walker for 30 min to cool them down. The racehorses were routinely exercised five times per week, and this training schedule was used throughout the entire race season. In accordance with the study’s protocol, the racehorses were submitted to tests twice, in May (MAY) and September (SEP), which coincided with the initial and end periods of the race season. The period between sampling was 18 weeks. On the day of the study, the horses were subjected to the same exercise loads as during routine ongoing training sessions. Blood samples were taken from each horse three times, always in the same manner. The first sample was taken at rest, in the early morning, before the training session, to determine all studied parameters; the second sample was taken immediately after the end of exercise, to determine biochemical indicators (LA, cortisol, plasma total proteins, AST, LDH); and the third was taken after 30 min of cooling down on an automatic horse walker, to measure post-exercise plasma irisin and myostatin concentrations. The studied horses did not show any clinical symptoms of any health disorders and mares did not showed external symptoms of estrus.

The second group comprised 10 endurance horses: Four mares, three stallions, and three geldings. Mean age of horses in this group amounted to 7.4 ± 1.95 years, including five 6-year-old horses and five older horses aged from 7 to 12 years (mean 8.8 ± 1.67). The horses were housed and trained individually in training centres throughout the country under the supervision of professional trainers, for at least 12 months. They were brought to the location of competitions at least a day before they started. In the middle of July, the horses were tested during national competitions covering 80 km (one-day competition according to rules of the International Federation for Equestrian Sports). This distance was chosen for the research because it is an average distance for endurance horses. Before the competition, all horses passed a pre-race obligatory veterinary inspection (data not shown). Only horses that completed the full distance were involved in the study. Blood samples were taken twice, namely at rest and immediately after covering the first 60 km of the distance, during the 30-min-rest before the final part of the competition. Because of technical reasons, sampling the blood 30 min after the end of exercise was not possible. The distance of 60 km was chosen for the study as it was long enough to induce changes in irisin and myostatin plasma concentration. It was feared that a finish after 80 km would cause micro-injuries in the ultra-structure of muscle fibers, which are the source of studied myokines, and, consequently, biased results. 

### 2.2. Blood Sampling and Analysis 

The blood samples were drawn by jugular venipuncture into vacuum tubes containing K2 EDTA (Vacutainer System, Becton-Dickinson Co., Franklin Lakes, NJ, USA). The collected samples were cooled down in a water bath to a temperature of 4 °C and then centrifuged to obtain plasma for further investigations. LA concentration in the fresh plasma samples collected at rest and immediately after exercise were determined by the enzymatic method (Cormay, Poland) using a Dr. Lange portable analyzer (Dr. Lange Laboratory System LP450, Dr. Lange, Berlin, Germany). The remaining plasma was frozen and stored at −74 °C for further biochemical analyses. The plasma concentration of myostatin was measured using a commercially available, equine-specific enzyme immunoassay GDF-8/Myostatin ELISA kit (R&D Systems Bio-Techne, Minneapolis, MN, USA) with a sensitivity of 2.25 pg/dL. The intra- and inter-assay CVs were <5.4% and <6.0%, respectively. Plasma irisin was estimated using the multi-species EIA kit (Phoenix Pharmaceuticals Inc., Burlingame, CA, USA), with a sensitivity of 6.8 ng/mL. The intra- and inter-assay CVs were <10.0% and <15.0%, respectively. 

For plasma cortisol determination, the CORTISOL ELISA kit (DRG International Inc., Mountainside, NJ, USA) was used. The intra- and inter-assay CVs for plasma cortisol concentration determined in the laboratory reached 6% and 8%, respectively. The values are expressed as ng/mL. For each myostatin, irisin, and cortisol test, absorbance was measured at 450 nm using a Multiskan reader (Labsystem, Helsinki, Finland) supported by GENESIS V 3.00 software (VWR International Ltd., Lutterworth, UK).

Plasma proteins concentration and activity of indicator enzymes: Aspartate aminotransferase (AST) and lactate dehydrogenase (LDH) were determined using enzymatic kits (Cormay, Warsaw, Poland) and Dr. Lange analyzer.

All samples were tested in duplicate in a blinded manner.

### 2.3. Statistical Analysis

Obtained data were tested for normality of distribution using the Shapiro-Wilk method. A normal distribution was confirmed. Thus, two analyses of variance were performed with SAS (Statistical analysis System, version 9.4, SAS Institute, Cary, NC, USA) using the Mixed model. For data obtained from racehorses, analysis of variance for repeated measurements was used with random effect of the horse and fixed effects of the period of the study (repeated factor: MAY and SEP), the time of blood sampling (at rest and after the end of exercise), the sex of the horse (stallions vs. mares), and their interactions. The models were reduced because the sex effect and its interactions with other analyzed factors were found statistically insignificant. Therefore, in the case of endurance horses, in view of low number of horses representing a given sex (four mares, three stallions, and three geldings), the data were treated as sex-independent also. Thus, for endurance horses, the considered factors were random effect of the horse and fixed effects of the time of blood sampling (at rest and immediately after the end of investigated exercise), the age of horses (6-year-old and older horses), and their interactions. The results are presented as last squares means (LSM) with standard errors (SE). The level of probability was accepted at 95% (*p* < 0.05). Differences between levels of analyzed effects were tested using the post-hoc multiple comparison for LSM. 

## 3. Results 

Results of variance analysis are shown in Table 2. Each analyzed parameter was influenced by at least one of the analyzed factors. In racehorses, exercise induced a statistically significant increase in plasma LA concentrations (Table 3). Other determined biochemical parameters did not differ significantly (data not showed). Values obtained immediately after exercise were higher in MAY than in SEP (*p* ≤ 0.05). In endurance horses, the values of plasma cortisol, total proteins, AST, and LDH increased significantly in response to exercise (Table 4).

**Figure 1:**
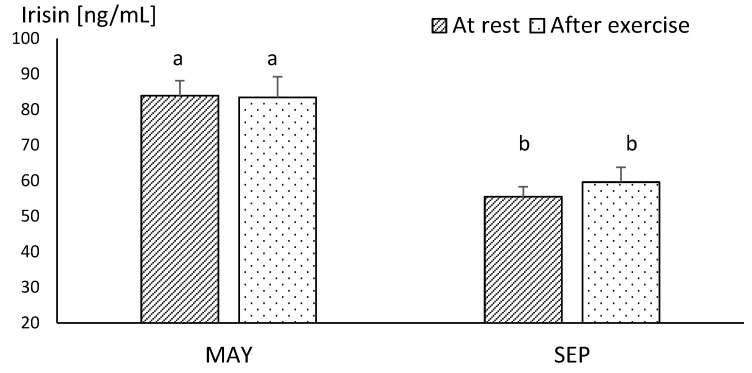
The mean values of plasma irisin concentration in racehorse at the beginning (MAY) and in the end (SEP) of the training season. Error bars represent standard errors (*n* = 10). a,b—different letters indicate significant differences (*p* ≤ 0.001).

**Figure 2:**
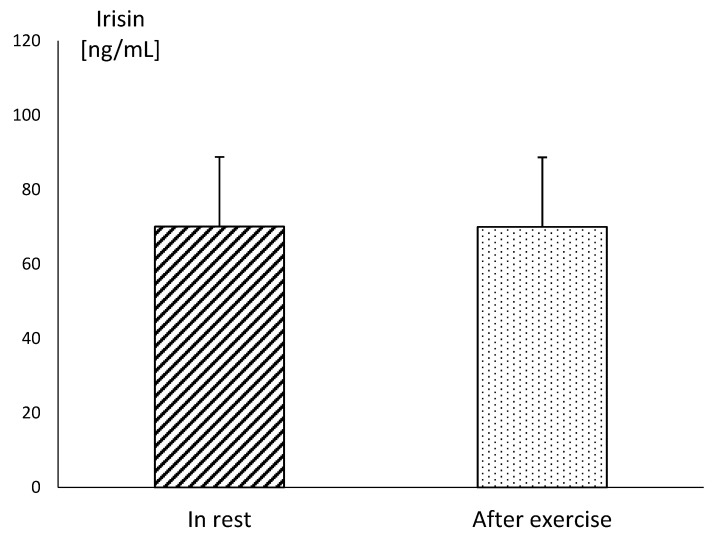
The mean values of irisin concentration in blood plasma in endurance horse in rest and after the end of the run at the distance of 60 km. Error bars represent standard errors (*n* = 10).

In MAY, the mean resting concentration of irisin was at a very similar level to the values obtained after exercise. There were no statistical differences between these values, although the mean concentration of irisin assessed at this time reached the highest level during the study. In SEP, the mean concentration of this myokine showed significantly lower resting values, in comparison to those obtained in MAY (*p* ≤ 0.001). Similarly, the post-exercise level of irisin was significantly lower (*p* ≤ 0.001) in SEP compared to its concentration in MAY (Figure 1). The plasma irisin concentration in endurance horses acquired immediately after running the race did not differ significantly in comparison to resting values (Figure 2). 

**Figure 3:**
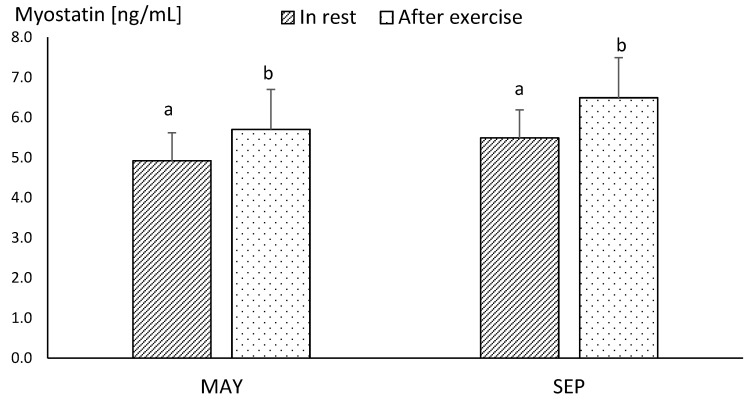
The mean values of plasma myostatin concentration in racehorse at the beginning (MAY) and in the end (SEP) of the training season. Error bars represent standard errors (*n* = 10). a,b—different letters indicate significant differences (*p* ≤ 0.05).

**Figure 4:**
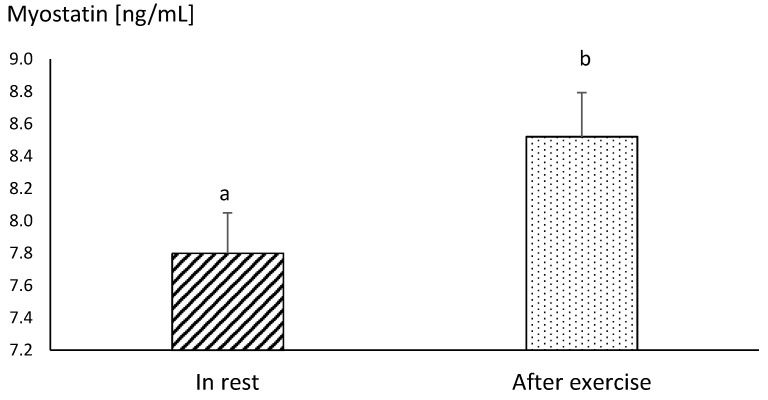
The mean values of myostatin concentration in blood plasma in endurance horses at rest and immediately after the end of the run at the distance of 60 km. Error bars represent standard errors (*n* = 10). a,b—different letters indicate significant differences (*p* ≤ 0.01)

Differences between the concentrations of myostatin obtained in MAY and SEP were statistically insignificant, both before and after exercise (Figure 3). The mean resting concentration of myostatin in endurance horses was significantly higher in comparison to the values obtained in racehorses, in both MAY and SEP (*p* ≤ 0.05) (Figure 3 and Figure 4). 

The post-exercise concentration of myostatin increased significantly in both racehorses and endurance horses, in comparison to the resting values (Figure 3 and Figure 4).

## 4. Discussion

The studied race and endurance horses performed two extremely different types of effort. High exercise-induced increase in LA value in racehorses, especially in MAY, means that the exercise performed by racehorses was anaerobic and was much more intense than that in endurance rides [45]. Moderate increase in plasma proteins, cortisol, AST, and LDH values in endurance horses indicated that the workload performed was typical for this kind of exercise and the horses were not excessively exhausted [45,46]. 

The present study shows that a single bout of exercise had no effect on plasma irisin concentration, regardless of the type of effort. However, it was stated that in racehorses, training led to a decrease in the resting plasma irisin concentration. As described by Rodriguez et al. [8], long-term exercise training negatively influences the secretion of this myokine into the bloodstream. This observation was confirmed in our study, namely a significantly lower concentration of irisin was found in SEP, at the end of the training season, compared with MAY. 

Recent studies have not demonstrated a clear effect of training on the circulating level of irisin. For example, in some reports, physical training in humans induced a decrease in circulating irisin [24,47,48], whereas other researchers noted no effect [22,49], or even an increase in its concentration [50]. Similarly ambiguous results have been reported in studies on trained rats [51,52,53,54,55]. However, these studies differed in various factors, such as the type of exercise used for training, the duration of training, as well as the body condition, sex, health status, etc. of the investigated subjects. Therefore, no direct comparison of the results obtained in our study with the reports cited above can be made. On the basis of our results, it can only be stated that intensive training of young horses lasting at least 18 weeks induced a decrease in plasma irisin concentration.

The effect of a single bout of exercise is also not clear. In our study, plasma irisin concentrations determined before and after exercise did not differ significantly. Similar results were obtained in humans who were investigated within 30 min after an exercise test [56,57,58]. However, other reports showed that in some subjects, the release of irisin increased in response to exercise [58,59,60]. A detailed analysis of the time-course of the plasma irisin concentration shed new light on this issue. Namely, in exercised humans, there is a time-point at which the level of irisin is similar to its resting values. This point appears 2–3 h after exercise, independently of the intensity of exercise performed. Then, within the next 6 to 19 h, after the end of exercise, the plasma irisin concentration increases [61]. Based on these observations, it can be assumed that the measurement of plasma irisin concentration at least 30 min after the end of exercise was performed too early to reveal any changes in its level. It follows that increasing the frequency of blood sampling should be considered in further studies. However, determining exercise-induced changes in plasma cytokine levels in blood samples collected 30 min after the end of short-term exercise is routinely used in horse studies [62,63]. Irisin and myostatin are proteins, in which synthesis is time-consuming processes but their half-life in blood plasma can be counted in hours. Therefore, changes in exercise-induced levels of cytokines measured immediately after the end of effort could be undetectable. 

Another interesting observation concerning the influence of physical exercise on the circulatory level of irisin was reported by Huh et al. [64]. The authors suggest that the post-exercise irisin concentration depends on the general fitness level of exercising horses, as they found a significant difference between the pre- and post-exercise level of circulating irisin only in the first week of training. After eight weeks of training, there were no significant differences. Both measurements were performed 30 min after finishing exercise. Hence, the lack of exercise-induced differences in the plasma irisin level in our study could be due to the fact that the horses were studied at least two months after starting the training process. 

The plasma concentration of myostatin, the other myokine studied, significantly increased in response to intensive short-term and endurance exercises in the studied horses. There is a lack of similar studies performed on horses with which to compare the results. In humans, the results of several studies are not conclusive in this respect. For example, circulating myostatin increased in response to acute exercise in young men [65], whereas it decreased in muscle bioptates collected after a single bout of acute exercise [66]. One of the possible explanations for the exercise-induced increase in plasma myostatin observed in our study is acidification of the horses’ muscle tissue during acute effort. A build-up of lactic acid in the muscles in response to exercise may promote myostatin release from muscle fibres and, as a consequence, increase its concentration in the bloodstream [26]. Based on the LA results obtained in our study, this supposition seems highly probable. 

Resting values of myostatin determined in MAY and SEP did not differ significantly. This means that training did not influence plasma myostatin in racehorses. In contrast, training of human and rat subjects generally led to a decrease in the serum/plasma myostatin concentration [49,67,68,69]; however, a physical exercise intervention in recumbent patients resulted in an increase in this myokine [70]. In turn, de Souza et al. [71] found no effect of different types of training on myostatin gene expression in muscles. Analogously, the plasma myostatin concentration analyzed in the present study seemed to be independent of the training level, because similar values were recorded in May and in September, within the same training season.

It is known that the level of myostatin depends on age [72]. The results of our study showed that the highest resting values of myostatin were recorded in endurance horses, which were older than the racehorses. In humans, higher values are linked with aging [72]. Data concerning horses indicate that only circulating myostatin level declines in geriatric horses [73]. Thus, this statement is not entirely consistent with our study. The horses involved in our study were at their prime age. Further studies are required for detailed explanation of the influence of age on plasma myostatin concentration in horses.

Whereas plasma irisin decreased in response to race training, myostatin remained unchanged, but a single bout of exercise induced an increase in plasma myostatin in both studied groups, while irisin remained at a constant level. In light of these findings, we suggest using of myostatin and irisin as a new markers in the assessment of effort efficiency in trained horses, however, after verifying our results on a larger group of tested horses. It has previously been reported that these two myokines inhibit each other’s synthesis and release [24]. Huh et al. [74] stated that elevation of circulating irisin stimulated muscle growth through the suppression of myostatin. An in vitro study revealed similar results [75]. In turn, myostatin negatively regulated irisin expression in skeletal muscles and its secretion into the bloodstream [32]. Conversely, the lack of myostatin stimulates irisin synthesis [35]. Moreover, myostatin inhibition stimulates fatty acid oxidation in muscle as well as the expression of mRNA encoding PGC-1α [76]. Protein PGC-1α stimulates not only mitochondria biogenesis and more efficient fatty acid oxidation but also irisin mRNA expression [35]. Particularly important is that myostatin promotes the remodeling of muscle tissue to increase the expression of the slow myosin heavy-chain isoform and inhibits the formation of the fast chain isoform (type II, anaerobic) [77,78]. In this context, the increase in plasma myostatin in response to exercise in horses could have a negative impact on the increase in the fast myosin heavy chain isoform, thereby protecting the organism against the overproduction of lactic acid. However, the scope of our study does not allow to support this hypothesis.

Finally, it is worth mentioning one more important function of irisin and myostatin. They both play a significant role in bone metabolism as factors influencing bone microstructure and mechanical properties [79]. Irisin plays a crucial role in enhancing bone remodelling and increasing cortical bone mass in experimental animals, which ultimately increases bone strength [23,80]. In contrast, myostatin exerts negative effects on bone mass through osteoclast activation, which leads to increased bone remodeling, where bone resorption outpaces bone formation [81]. From the exercise physiology point of view, bone and muscle form one functional system and are tightly connected to each other. Thus, understanding of these interactions seems to be particularly important in sport horses due to the high training loads to which they are subjected daily. Further studies with higher amount of animals trained on different distances and different ages are needed.

Although the present study has yielded some preliminary findings, its design is not without limitations. This study was focused on the exercise as a general factor influencing the plasma irisin and myostatin levels in trained horses. However, there might be some relevant factors that additionally influenced on the concentration of irisin and myostatin in horses, for example horses’ type of use, and maintaining routine. Moreover, there is the lack of similar studies in a horse, hence it was not possible to discuss our results with others. Therefore, this preliminary study should be considered with caution, and further research is needed to explain the regulation of irisin and myostatin release in exercised horses.

## 5. Conclusions

The present preliminary study demonstrates that the plasma irisin concentration decreased in response to training in racehorses. Physical effort strongly increased the plasmatic myostatin level in horses, trained for both race and endurance competitions. Simultaneously, physical exercise did not influence the concentration of circulating irisin, which may suggest that myostatin released in response to exercise provides a negative feedback signal to irisin release

## Figures and Tables

**Table 1:** Technical parameters of the exercise performed by the horses (means ± SD).

Horses		*n*	Distance	Average Velocity	Average Duration
Racehorses	MAY	10	1200 m	9.97 ± 0.25 m/s	2.0 ± 0.05 min
	SEP	10	1200 m	9.01 ± 0.22 m/s	2.2 ± 0.06 min
Endurance horses		10	60 km	3.33 ± 0.06 m/s	300 ± 5.15 min

MAY, SEP—stages of training season (see the text for details).

**Table 2:** Results of variance analysis.

Parameter	Racehorses			Endurance Horses		
	Factor	F	*p*	Factor	F	*p*
Irisin	Time	0.03	0.9436	Time	1.33	0.2796
	Period	6.62	**0.0008**	Age	1.83	0.1368
	Interaction	1.98	0.1687	Interaction	0.43	0.7821
Myostatin	Time	3.81	**0.0329**	Time	7.91	**0.0016**
	Period	0.81	0.4544	Age	0.89	0.4167
	Interaction	2.06	0.1376	Interaction	1.80	0.1403
Lactic acid	Time	19.91	**0.0001**	Time	2.45	0.1024
	Period	7.40	**0.0023**	Age	1.33	0.2791
	Interaction	3.59	**0.0392**	Interaction	0.76	0.3613
Cortisol	Time	2.35	0.0931	Time	14.53	**0.0006**
	Period	0.44	0.7821	Age	2.67	0.0843
	Interaction	1.21	0.2971	Interaction	1.28	0.3110
Proteins	Time	1.53	0.1138	Time	5.18	**0.0126**
	Period	0.47	0.4992	Age	1.33	0.2790
	Interaction	0.81	0.4543	Interaction	0.95	0.7803
AST	Time	1.24	0.3011	Time	4.39	**0.0034**
	Period	1.43	0.2503	Age	0.67	0.5167
	Interaction	0.85	0.3451	Interaction	0.13	0.8705
LDH	Time	1.80	0.1400	Time	15.38	**0.0002**
	Period	1.38	0.2119	Age	1.27	0.3083
	Interaction	0.08	0.8906	Interaction	1.68	0.1907

Time—time of blood sampling (at rest vs. after exercise), Period—period of the study (May vs. SEP), Age—age of endurance horses (6-year-old vs. older), Interaction—interaction between the factors mentioned above, F—value of the Fisher–Snedecor test; *p* values in bold are statistically significant.

**Table 3:** Plasma lactic acid concentration in studied racehorses (lsm ± SE).

Time of the Test	At Rest	Immediately after the End of Exercise
MAY	0.94 ± 0.02 ^a^	9.94 ± 1.27 ^b^
SEP	1.03 ± 0.01 ^a^	2.68 ± 0.91 ^c^

^a,b,c^—different letters indicate significant differences.

**Table 4:** Biochemical parameters determined in studied endurance horses (lsm ± SE).

Parameter	At Rest	After the End of Exercise
Lactic acid (mmol/L)	0.82 ± 0.01	1.46 ± 0.12
Cortisol (ng/mL)	152 ± 5.08	237 ± 7.07 *
Total proteins (g/L)	61.4 ± 3.31	65.3 ± 2.43 *
AST (U/L)	235 ± 19.4	263 ± 16.2 *
LDH (U/L)	458 ± 33.7	698 ± 60.4 *

*—significantly higher than obtained at rest.
